# *Plasmodium ovale wallikeri* and *P. ovale curtisi* Infections and Diagnostic Approaches to Imported Malaria, France, 2013–2018

**DOI:** 10.3201/eid2702.202143

**Published:** 2021-02

**Authors:** Valentin Joste, Justine Bailly, Véronique Hubert, Cécile Pauc, Mathieu Gendrot, Emilie Guillochon, Marylin Madamet, Marc Thellier, Eric Kendjo, Nicolas Argy, Bruno Pradines, Sandrine Houzé, on behalf of the French National Reference Center for Imported Malaria Study Group

**Affiliations:** Centre National de Référence du Paludisme, Paris, France (V. Joste, J. Bailly, V. Hubert, C. Pauc, N. Argy, S. Houzé);; Université de Paris, Paris (N. Argy, S. Houzé);; Laboratoire de Parasitologie-Mycologie, Paris (V. Joste, N. Argy, S. Houzé);; Institut de Recherche Biomédicale des Armées, Marseille, France (M. Gendrot, M. Madamet, B. Pradines);; Aix–Marseille Université, Marseille (M. Gendrot, M. Madamet, B. Pradines);; Instituts Hospitalo–Universitaires Méditerranée Infection, Marseille (M. Gendrot, M. Madamet, B. Pradines);; Centre National de Référence du Paludisme, Marseille (M. Madamet, B. Pradines);; Sorbonne Université, Paris, France (M. Thellier, E. Kendjo)

**Keywords:** *Plasmodium ovale curtisi*, *Plasmodium ovale wallikeri*, imported malaria, diagnosis, malaria-endemic countries, parasites, France, malaria

## Abstract

We retrospectively analyzed epidemiologic, clinical, and biologic characteristics of 368 *Plasmodium ovale wallikeri* and 309 *P. ovale curtisi* infections treated in France during January 2013–December 2018. *P. ovale wallikeri* infections displayed deeper thrombocytopenia and shorter latency periods. Despite similar clinical manifestations, *P. ovale wallikeri*–infected patients were more frequently treated with artemisinin-based combination therapy. Although the difference was not statistically significant, *P. ovale wallikeri*–infected patients were 5 times more frequently hospitalized in intensive care or intermediate care and had a higher proportion of severe thrombocytopenia than *P. ovale curtisi*–infected patients. Rapid diagnostic tests that detect aldolase were more efficient than those detecting *Plasmodium* lactate dehydrogenase. Sequence analysis of the *potra* gene from 90 *P. ovale* isolates reveals an insufficient polymorphism for relapse typing.

## Abstract 2

Patients infected with *P. ovale wallikeri* displayed deeper thrombocytopenia and a shorter latency period.

Malaria is a vectorborne disease caused by *Plasmodium*, a parasite transmitted by *Anopheles* mosquitoes. In 2018, malaria was responsible for ≈228 million cases and 405,000 deaths worldwide (*1*). *Plasmodium ovale* is endemic in Africa and represents the main agent of relapsing malaria (*2*). In mainland France, *P. ovale* was responsible for ≈6% of imported malaria cases in 2018 (*3*). Since the 2017 Frances updates for *Plasmodium* infection management recommendations, first-line treatment of *P. ovale* infections is based on chloroquine- or artemisinin-based combination therapy (ACT), instead of atovaquone/proguanil (*4*).

Because of low parasite density and poor efficiency of rapid diagnostic test (RDT) detection (*5*), *P. ovale* infections are difficult to diagnose. Consequently, infections caused by *P. ovale* remain poorly studied, and little is known about the global burden of the disease worldwide or its geographic distribution.

Since 2010, *P. ovale* has been divided into 2 species, *Plasmodium ovale wallikeri* and *P. ovale curtisi*, on the basis of gene polymorphisms (*6*–*8*). *P. ovale wallikeri* appears to cause malaria infections with a shorter latency period (*9*,*10*) and with deeper thrombocytopenia than *P. ovale curtisi* (*11*,*12*). Both *P. ovale wallikeri* (*13*) and *P. ovale curtisi* (*14*) can be responsible for a clinical relapse event, defined as renewed asexual parasitemia originating from liver dormancies (*2*). Relapse characterization relies on microscopic diagnosis and medical history. No consensus molecular method for *P. ovale* spp. relapse typing is reported. However, *P. ovale* tryptophan–rich antigen (*potra*) gene sequencing has previously been used for genotyping purpose (*13*,*14*).

At the microscopic level, the only observable difference between the species is a lack of Schüffner granulations in *P. ovale wallikeri* infected erythrocytes (*15*). However, this feature is rare and difficult to see, which makes *P. ovale* species distinction almost impossible even for an experienced microscopist. Molecular biology is a promising tool and is both sensitive and specific for the differentiation of *P. ovale wallikeri* from *P. ovale curtisi*. The first nested PCR that discriminates *P. ovale wallikeri* and *P. ovale curtisi* was developed in 2007 (*16*), and the first quantitative PCR (qPCR) was developed in 2013 (*17*).

In this study, we conducted a large retrospective multicenter analysis of imported *P. ovale* cases. Epidemiologic, clinical, and biologic characteristics of 309 *P. ovale curtisi*– and 368 *P. ovale wallikeri*–infected patients treated in France during January 2013–December 2018 were analyzed. The effectiveness of Rapid Diagnostic Test (RDT) and the polymorphism of *potra* gene were also investigated.

## Methods

### Sample Selection

France’s National Malaria Reference Center (FNMRC) is in charge of epidemiologic surveillance of imported malaria in France. Whole blood samples of patients with *Plasmodium* infections were received from hospital correspondents in France. FNMRC correspondents also reported demographic, epidemiologic, clinical, and biologic data through a reporting website. We retrospectively selected all the reported and PCR-confirmed *P. ovale* infections that occurred during January 2013–December 2018.

### DNA Extraction

DNA was extracted from 200 μL of whole blood samples by using Magnapure automaton (Roche Diagnostics, https://diagnostics.roche.com) and eluted in 100 μL of elution buffer, according to the manufacturer’s instructions. DNA was stored at –20°C until further analysis.

### Diagnosis of *P. ovale* Infection

The diagnosis of *Plasmodium ovale* infection was made by the hospital correspondent and confirmed by FNMRC with a thin blood smear reading, a thick blood smear reading, or both. Thick blood smears were considered positive if >1 trophozoïtes was visualized after examination of 1,000 leukocytes. Thin blood smears were used to confirm *Plasmodium* species identification. Parasite density was calculated by using the formula parasite density (parasites per μL) = patient leukocyte count (per µL) × (no. parasites counted)/(no. leucocytes counted), according to World Health Organization (WHO) recommendations (*18*). Parasitemia was calculated by counting the percentage of infected red blood cells on thin blood smears according to WHO recommendations (*18*). All *P. ovale* infections were confirmed with nested PCR (*19*,*20*) during 2013–2014, with qPCR–Taqman (Launch Diagnostics, https://www.launchdiagnostics.com) during 2015–2017, and with Bio-Evolution (https://www.bio-evolution.net/index.php) in 2018.

### *P. ovale curtisi* and *P. ovale wallikeri* differentiation

qPCR–high-resolution melting (HRM) targeting the 18S rRNA gene was performed to differentiate *P. ovale wallikeri* from *P. ovale curtisi* by using Plasmo1_F and Plasmo2_R primers. The method development and validation was described previously (*21*). In brief, qPCR-HRM results were compared with nested PCR results from Calderaro et al. (*16*), and they displayed similar species determination. In all studied samples, *P. ovale wallikeri* and *P. ovale curtisi* melting plots displayed 2 specific melting temperatures (Tm) as Tm_1_ and Tm_2_, and the ΔTm between the 2 Tm was calculated.

For uncertain results (i.e., only 1 Tm on melting plot analysis [*21*]), nested PCR was performed by using rPLU1 and rPLU5 primers in the first PCR reaction and rOVA1/rOVA2 for *P. ovale curtisi* amplification or rOVA1v/rOVA2v for *P. ovale wallikeri* amplification in second PCR reaction (*16*). PCR products were visualized on 1% agarose gel stained with GelRed (https://biotium.com). We used *P. ovale wallikeri* and *P. ovale curtisi* isolates as positive controls and water as a negative control for each qPCR-HRM run.

### RDT Efficiency in *P. ovale wallikeri* and *P. ovale curtisi* Detection

We evaluated the efficiency of 4 different RDTs detecting pan-*Plasmodium* proteins (aldolase or *Plasmodium* lactate dehydrogenase [pLDH]) for the detection of *P. ovale wallikeri* and *P. ovale curtisi*. Vikia Malaria Ag Pf/Pan (bioMérieux, https://www.biomerieux.com) (*22*) and Binax Now Pf/Pan (Abbott, https://www.abbott.com) (*23*) were used for aldolase detection (aldolase-RDT). Palutop+4 Pan/Pv/Pf (Biosynex, https://www.biosynex.com) (*24*) and Core Malaria Pan/Pv/Pf (Core Diagnostics, https://www.corediagnostics.net) were used for pLDH protein detection (pLDH-RDT). Results were interpreted according to the manufacturer’s instructions.

### Data Collection

Each hospital correspondent sent an EDTA blood sample of a patient infected with *P. ovale* to FNMRC. This process was completed by using the online patient form containing multiple data, including demographic data (place of birth, ethnicity, age, and sex), epidemiologic data (trip purpose, visited country, duration of travel, and use of prophylaxis or bed nets), biologic data (parasite count, RDT results, leukocytes, hemoglobin and platelet counts, with severe thrombopenia defined as <50 G/L [*25*], and date of diagnosis), and clinical data (date of symptom onset, fever, headache, asthenia, and arthralgia or myalgia, as well as free symptomatology description for other symptoms, antimalarial treatment used, hospital or ambulatory regimen, and duration of hospitalization). Severe malaria biologic and clinical signs, adapted from the severe *P. falciparum* WHO recommendations (*4*,*26*), and relapsing *P. ovale* infection, defined as new *P. ovale* infection after a first completed and effective antimalarial treatment (*27*), were reported.

The latency period was calculated for each infection by subtracting the date of return from travel to the onset of the symptoms as defined by Rojo-Marcos et al. (*11*,*12*). The period of high malaria transmission in West Africa was defined as August–November on the basis of Nabarro et al. definition (*10*). The delay between symptom onset and diagnosis was also determined. We looked for false or incomplete microscopic diagnosis (*Plasmodium* spp.) to estimate the potential effect on *P. ovale* microscopic diagnosis of the described lack of Schüffner granulations in *P. ovale wallikeri*–infected erythrocytes (*15*).

No specific consent was required from patients because the parasitologic data were collected from the FNMRC database and analyzed in accordance with the common public health mission of all National Reference Centers in France, in coordination with the Santé Publique France organization for malaria surveillance and care. The study of the biologic samples obtained from routine medical care was considered as noninterventional research accordingly to article L1221–1.1 of the public health code in France and only requires the nonopposition of the patient during sampling (per article L1211–2 of the public health code). All data collected were anonymized before analysis.

### *potra* Sequencing and Analysis

We amplified *potra* fragments as previously described (*28*). Bidirectional sequencing reaction was performed for the secondary *potra* fragment. Gene sequences were analyzed with Sequencher 5.0 (Genecodes, http://www.genecodes.com). Isolates from GenBank under accession nos. HM594183 (*28*), MG588152, and MG588154 (*29*) were used as *P. ovale curtisi* reference sequences; HM594180 (*28*) and MG588148–150 (*29*) were used as *P. ovale wallikeri* reference sequences.

### Statistical Analysis

*P. ovale wallikeri* and *P. ovale curtisi* infections were compared in terms of demographic, epidemiologic, clinical, and biologic characteristics. The Kolmogorov-Smirnov test with the Lilliefors correction was used to verify the normality of variables distributions, and the Levene test was used to verify the homogeneity of the variances. If both criteria were validated, a Student *t*-test was used; otherwise, a Mann-Whitney U-test was performed to compare medians. Proportions were compared by using the χ^2^ or Fisher exact test according to sample size (>5 or <5). R software was used to perform statistical tests (*30*).

## Results

### *P. ovale* Sample Selection 

**Figure 1:**
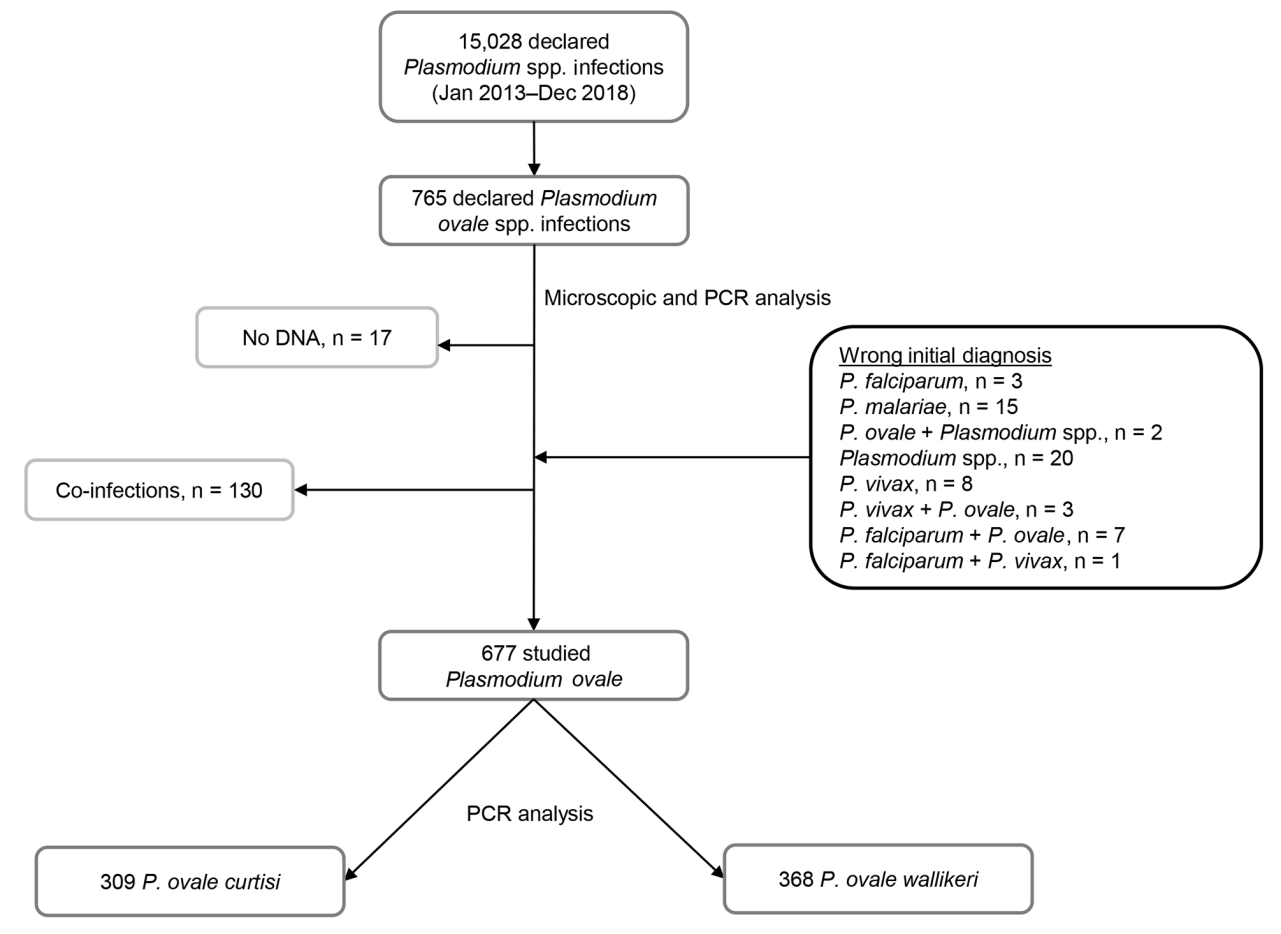
Flow-chart of the retrospective study analyzing characteristics of *Plasmodium ovale wallikeri* and *P. ovale curtisi* infections treated in France during January 2013–December 2018. All reported *P. ovale* infection cases were confirmed with microscopy and PCR analysis, and co-infections were excluded. A total of 59 *P. ovale* isolates initially misdiagnosed by the hospital correspondent were added. A total of 677 *P. ovale* infection cases were included in the study.

During January 2013–December 2018, 15,028 *Plasmodium* spp. infection cases were reported to NMRC, including 765 *P. ovale* infections. Seventeen cases were excluded from the analysis because blood sample were unavailable. After exclusion of co-infections and inclusion of 59 *P. ovale* initially misdiagnosed (confirmed by PCR), 677 *P. ovale* cases from 63 different hospitals in France were finally included (Figure 1). By using qPCR-HRM for species differentiation, we identified 368 *P. ovale wallikeri* and 309 *P. ovale curtisi* infections. The 2 species segregated perfectly in qPCR-HRM; *P. ovale wallikeri* had a ΔTm of 1.62–2.69, and *P. ovale curtisi* had a ΔTm of 2.84–4.22.

### Patients’ Demographic and Epidemiologic Characteristics

**Figure 2:**
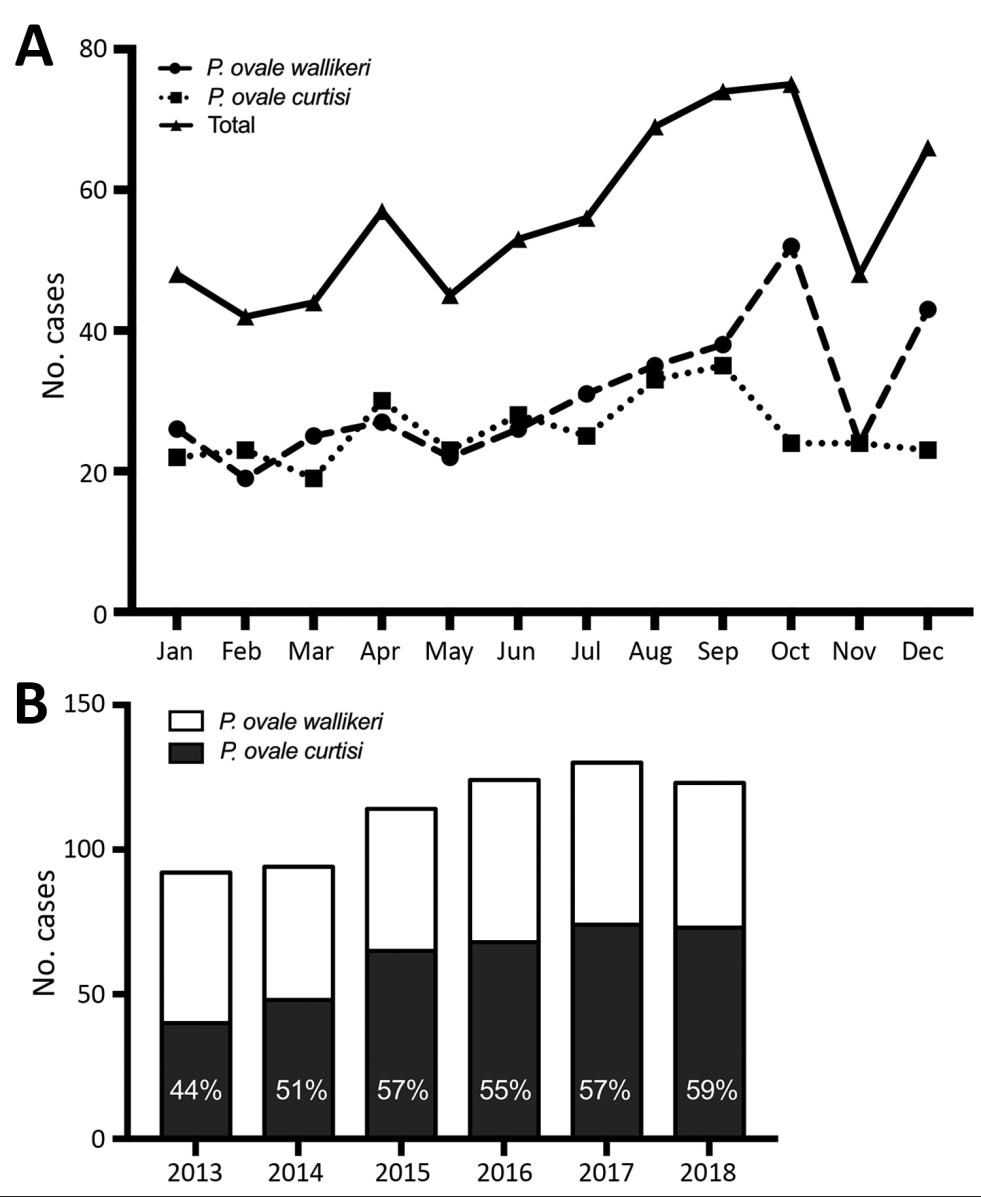
Number of *Plasmodium ovale* infection cases included in a study analyzing characteristics of *P. ovale wallikeri* and *P. ovale curtisi* infections treated in France during January 2013–December 2018, by month of inclusion (A) and year of inclusion (B).

*P. ovale wallikeri* and *P. ovale curtisi* showed similar repartition by month, except for October, which showed an increase in *P. ovale wallikeri* infections and a decrease in *P. ovale curtisi* cases (Figure 2, panel A). Among *P. ovale* cases, the proportion of *P. ovale wallikeri* infections increased from 44% to 59% during January 2013–December 2018 (Figure 2, panel B).

**Figure 3:**
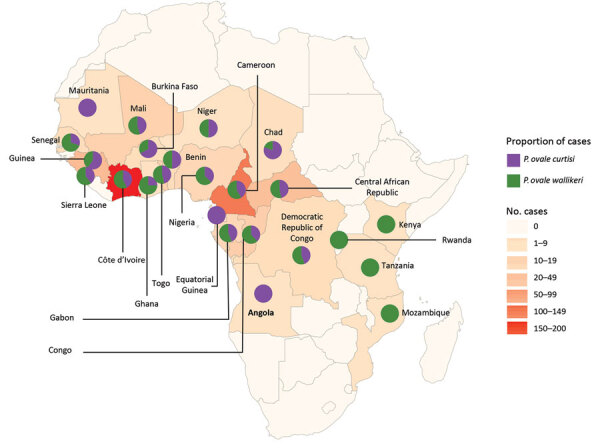
Geographic repartition of the origin countries of imported *Plasmodium ovale wallikeri* and *P. ovale curtisi* infection cases into France, January 2013–December 2018. Pie charts showed the repartition of cases between both species in each country.

*P. ovale wallikeri*– and *P. ovale curtisi*–infected patients did not display any differences in demographic and epidemiologic characteristics (Table 1). Countries of contamination were not statistically different between imported *P. ovale curtisi* and *P. ovale wallikeri* cases (p = 0.52) (Figure 3; Appendix Table 1).

**Table 1:** Demographic and epidemiologic characteristics of patients infected with *Plasmodium ovale wallikeri* and *P. ovale curtisi*, France, January 2013–December 2018*

Characteristic	*P. ovale curtisi*, n = 309	*P. ovale wallikeri*, n = 368	p value
Age, y, median (IQR)	31 (21–47)	34 (21–47)	0.973
Sex, %			0.716
M	63.4	61.4	
F	36.6	38.6	
Ethnicity			0.502
Black	200 (74.3)	239 (75.7)	
White	64 (23.8)	68 (21.5)	
Asian	2 (0.7)	1 (0.3)	
Other	3 (1.2)	8 (2.5)	
If African, place of birth			0.420
Africa	144 (83.2)	164 (80)	
Nonendemic country	29 (16.8)	41 (20)	
Type of patient			0.192
Immigrant†	23 (11.6)	21 (8.6)	
Traveler‡	137 (68.8)	187 (77.3)	
Visiting friends or relatives	109 (79.6)	152 (81.3)	
Tourism	6 (4.4)	8 (4.3)	
Work	22 (16)	27 (14.4)	
Resident	19 (9.5)	20 (8.3)	
Expatriate	6 (38.6)	10 (50)	
Humanitarian	13 (61.4)	10 (50)	
Military	20 (10.1)	14 (5.8)	
Duration of travel, d, median (IQR)	58 (29–91)	50 (24–91)	0.106
Chemoprophylaxis			0.882
Yes	97 (40)	123 (39.3)	
Complete	35 (44.9)	42 (43.8)	
Incomplete	43 (55.1)	54 (56.2)	
Prematurely stopped	26 (60.5)	36 (66.7)	
Occasionally taking	17 (39.5)	18 (33.3)	
No data	19 (NA)	27 (NA)	
No	146 (60)	190 (60.7)	
Using bed nets			0.119
Yes	48 (26.7)	41 (20.2)	
No	130 (73.3)	162 (79.8)	

*Values are no. (%) patients except as indicated. IQR, interquartile range; NA, not available. †A person who was born and lived in Africa. ‡A person who lived in a non–*Plasmodium*-endemic country.

For well-followed chemoprophylaxis (n = 77), the main treatments used were doxycycline (48%), atovaquone/proguanil (25%), and mefloquine (18%). No statistically significant differences were observed in the percentage of infection between those treatments.

### *P. ovale* Diagnosis

Parasite densities for *P. ovale curtisi* and *P. ovale wallikeri* infections were similar (median 4,500 parasites/µL, [interquartile range (IQR) 1,094–10,197 parasites/µL] for *P. ovale curtisi* vs. median 3,970 parasites/µL [IQR 598–9,240 parasites/µL] for *P. ovale wallikeri*). We noted 8.5% of species misidentification for *P. ovale curtisi* and 9% for *P. ovale wallikeri* (Figure 1).

### Aldolase and pLDH-RDT Efficiency

**Figure 4:**
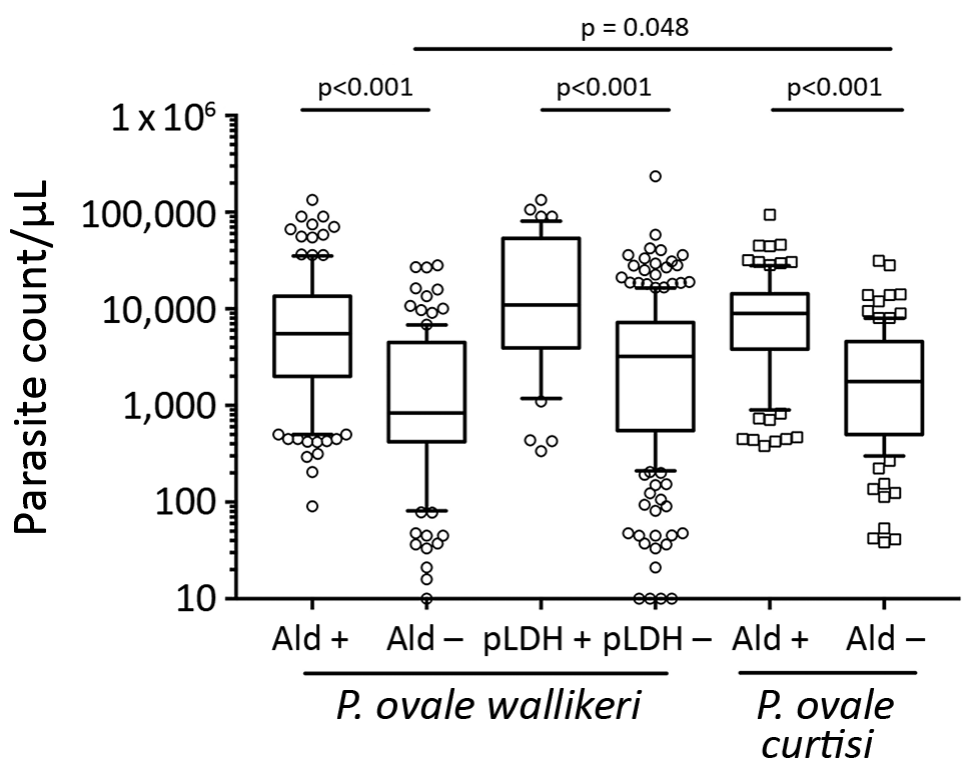
Comparison of parasite count according to RDT results in study analyzing characteristics of *Plasmodium ovale wallikeri* and *P. ovale curtisi* infections treated in France during January 2013–December 2018. Ald, aldolase RDTs; pLDH, plasmodium lactate dehydrogenase RDTs; Poc, *P. ovale curtisi*; Pow, *P. ovale wallikeri*; RDT, rapid diagnostic test.

We compared the diagnostic performance of aldolase-RDTs and pLDH-RDTs for *P. ovale* diagnosis. Aldolase-RDTs detection were more efficient in *P. ovale* spp. detection than pLDH-RDTs (p<0.001); no differences between the 2 species were observed. *P. ovale wallikeri* was more frequently detected with pLDH-RDT than *P. ovale curtisi* (p<0.001) (Table 2). The positivity of aldolase and pLDH-RDTs were strongly associated with parasite density. Percentage of positive RDT results increased with parasite density for both pLDH-RDT and aldolase-RDT (Table 2). A positive aldolase-RDT result was associated with a parasite density significantly higher than with a negative aldolase-RDT result for both species (median 6,612 parasites/µL [IQR 2,410–14,175 parasites/µL] for *P. ovale wallikeri* vs. median 1,287 parasites/μL [IQR 450–4,500 parasites/μL] for *P. ovale curtisi*; p<0.001) (Figure 4). Similarly, the parasite density of positive pLDH-RDT *P. ovale wallikeri* samples were significantly higher than those of negative pLDH-RDT (median 11,000 parasites/µL [IQR 3,960–52,910 parasites/µL] vs. median 3,227 parasites/µL [IQR 551–7,118] parasites/µL; p<0.001). Vikia (bioMérieux) aldolase-RDT had a greater accuracy for detecting *P. ovale* infections compared than did Binax Now (Abbott) (59.3% vs. 40.9%; p<0.001) and a better sensitivity (median 4,230 parasites/µL [IQR 1,205–9,450 parasites/µL] for positive Vikia vs. median 8350 parasites/µL [IQR 4,032–16,166 parasites/µL] for positive Binax Now; p<0.001).

**Table 2:** Comparison of aldolase and pLDH-RDT efficiency in *Plasmodium ovale wallikeri* and *P. ovale curtisi* infection diagnosis, France, January 2013–December 2018*

RDT result	Parasite density, parasites/μL	LDH				Aldolase		
		*P. ovale *	*P. ovale wallikeri*	*P. ovale curtisi*		*P. ovale *	*P. ovale wallikeri*	*P. ovale curtisi*
Positive		55 (10.6)	45 (16)	10 (4.2)		211 (47.8)	120 (50)	91 (45.3)
	<1,000	5 (3.9)	3 (3.9)	2 (3.9)		25 (19.5)	16 (20)	9 (17.6)
	1,000–5,000	15 (9.4)	14 (15)	1 (1.5)		65 (40.6)	42 (54.5)	23 (33.8)
	5,000–10,000	6 (7.8)	5 (12)	1 (2.8)		44 (57.1)	24 (66.7)	20 (57.1)
	10,000–50,000	16 (16.2)	11 (20)	5 (11.4)		67 (67.7)	29 (78.4)	38 (86.4)
	>50,000	13 (86.7)	12 (86)	1 (100)		10 (100)	9 (100)	1 (100)
Negative		465 (89.4)	237 (84)	228 (95.8)		230 (52.2)	120 (50)	110 (54.7)
p value		<0.001				0.322		

*Values are no. (%) patients except as indicated. LDH, lactate dehydrogenase; pLDH, plasmodium lactate dehydrogenase; RDT, rapid diagnostic test. †Proportions of positive and negative LDH or aldolase-RDT were compared for *P. ovale wallikeri* and *P. ovale curtisi* by using a χ^2^ test.

### Biologic and Clinical Characteristics

Patients infected with *P. ovale wallikeri* displayed deeper thrombocytopenia than those with *P. ovale curtisi* (Table 3), but reported symptomatology and disease severity did not differ. *P. ovale wallikeri* infections had shorter latency periods and a higher proportion of latency periods <50 days (p<0.001) (Table 3). Compared with patients who did not take prophylactic treatment, patients who reported well-managed prophylactic treatment had longer latency periods (median 90 days [IQR 47–177 days] vs. median 30 days [IQR 8–125 days]; p<0.001). Uncompleted prophylactic treatment did not extend latency period (median 33 days [IQR 17–112 days] vs. median 30 days [IQR 8–125 days]; p = 0.34). Military patients had longer latency periods than other patients (median 109 days [IQR 57–159 days] vs. median 40 days [IQR 12–142 days]; p = 0.0018), as did Caucasian versus African patients (median 84 days [IQR 28–140 days] vs. median 42 days [IQR 12–147 days]; p = 0.005 days). In the African population, no differences were found between African-born patients and others (mean 53 days [IQR 12–170 days] vs. mean 35 days [IQR 11–117 days]). The latency period was shorter in symptomatic patients returning from West Africa during the malaria season than in low-transmission or no-transmission seasons (median 27 days [IQR 10–67 days] vs. medina 90 days [IQR 17–158 days]; p<0.001) (Appendix Figure). *P. ovale wallikeri* infections and *P. ovale curtisi* infections were each responsible for 16 reported clinical relapses. 

**Table 3:** Biologic and clinical characteristics of *Plasmodium ovale wallikeri* and *P. ovale curtisi* infections, France, January 2013–December 2018*

Characteristic	*P. ovale curtisi*, n = 309	*P. ovale wallikeri*, n = 368	p value
Parasite density, parasites/µL, median (IQR)	4,500 (1,094–10,197)	3,970 (598–9,240)	0.112
Leucocyte count, G/L, median (IQR)	5.6 (4.4–7.1)	5.2 (4.1–6.5)	0.0501
Hemoglobin, g/L, median (IQR)	127 (113–140)	126 (114–139)	0.855
Platelet count, G/L, median (IQR)	111 (84–145)	94 (70–130)	<0.001
<75	56 (19.4)	104 (31)	
75–150	168 (58.1)	174 (51.9)	0.003
>150	65 (22.5)	57 (17.1)	
Severe thrombocytopenia	13 (4.5)	25 (7.5)	0.123
Diagnostic delay, d, median (IQR)	5 (3–7)	4 (2–7)	0.583
Delay between return from endemic country and onset of symptoms, d, median (IQR)	72 (18–208)	34 (10–95)	<0.001
<50 days	87 (42.4)	150 (59.5)	<0.001
Symptoms			
Fever	262 (95.6)	316 (97.8)	0.125
Arthralgia or myalgia	120 (54.8)	138 (57.7)	0.525
Asthenia	108 (58)	133 (61.3)	0.506
Headache	151 (68.6)	201 (75.3)	0.103
Anorexia	5	4	
Diarrhea	13	18	
Abdominal pain	28	29	
Nausea	16	20	
Vomiting	24	13	
Cough	6	12	
Clinical categorization			0.927
Uncomplicated malaria	293 (97.7)	335 (97.4)	
Severe malaria	3 (1)	5 (1.5)	
Asymptomatic	4 (1.3)	4 (1.1)	
Admission to hospital	158 (55.4)	196 (60.3)	0.243
Duration of hospitalization, d, median (IQR)	2 (1–3)	3 (1–4)	0.0732
Intensive- or intermediate-care hospitalization	1 (2.2)	7 (11.3)	0.134
Conventional hospitalization	46 (97.8)	55 (88.7)	
Treatment			0.00359
Chloroquine	147 (54.8)	152 (47.8)	
Artemisinin therapy	46 (17.1)	93 (29.2)	
Artemeter/lumefantrine	11 (25.5)	39 (41.9)	
Artesunate	2 (4.3)	5 (5.4)	
Arteminol/piperaquine	33 (70.2)	49 (52.7)	
Atovaquone/proguanil	64 (23.9)	64 (20.1)	
Mefloquine	3 (1.2)	0 (0)	
Quinine	8 (3)	9 (2.9)	

*Values are no. (%) patients except as indicated. IQR, interquartile range.

### Patient Care

A similar proportion of patients were hospitalized in the *P. ovale curtisi* and *P. ovale wallikeri* groups. Eight malaria case-patients with WHO-defined severe criteria (*26*) were reported during the period analysis (Table 3). *P. ovale wallikeri*–infected patients were 5 times more likely to be hospitalized in intensive or intermediate care than *P. ovale curtisi*–infected patients (Table 3). A higher percentage of *P. ovale wallikeri* infections were treated with ACT (29.2% vs. 17.1%; p<0.001), but no association was found between ACT treatment and parasite density, between ACT treatment and platelet count, or between ACT treatment and positive and negative RDTs. Patients treated with ACT did have shorter latency periods than other patients (median 33 days [IQR 11–111 days] vs. 54 days [IQR 15–170 days]; p = 0.025) and patients with latency periods <50 days were more often treated with ACT than others (28.6% vs. 20.3%; p = 0.048). This high proportion of ACT prescription was highest in patients with latency periods <50 days and platelet counts <60 G/L (52.3% vs. 22.7%; p = 0.002).

**Figure 5:**
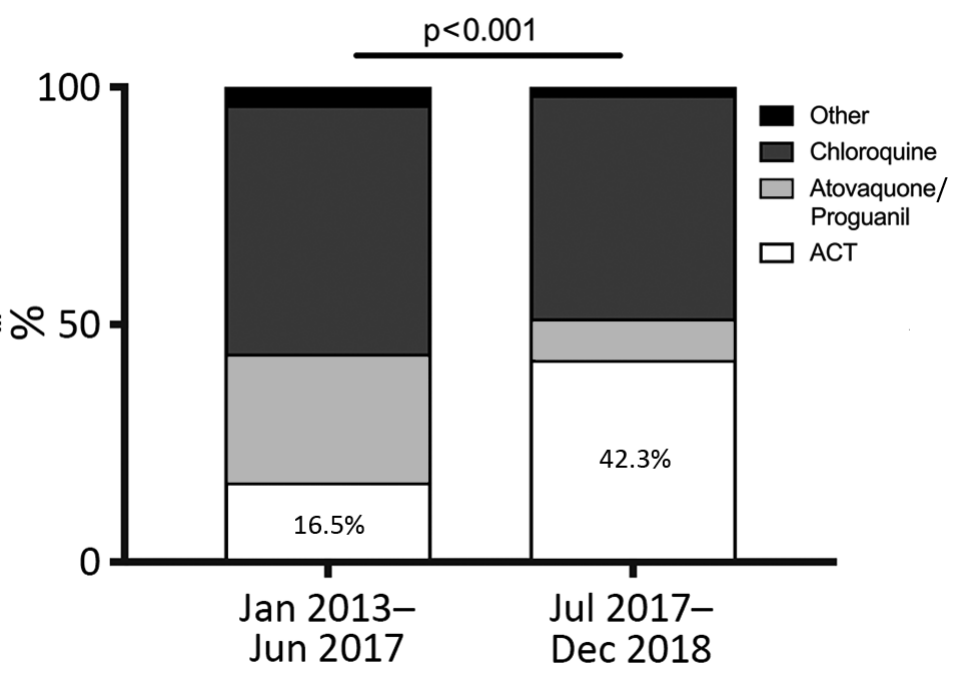
Effects of the new ACT treatment recommendations for *Plasmodium* spp. infections from La Société de Pathologie Infectieuse de Langue Française, revised in June 2017. ACT, chloroquine- or artemisinin-based combination therapy.

New recommendations from the Infectious Diseases Society in France (La Société de Pathologie Infectieuse de Langue Française) edited in 2017 (*4*) had a clear effect on *P. ovale* infection treatment (Figure 5), including replacement of atovaquone/proguanil by artemisinin-based combination therapy. However, little change in rates of chloroquine prescription occurred (52.5% before the revisions and 47.2% after).

For the period analyzed, no statistically significant relationship was found between the number of included *P. ovale* infection cases per hospital and the percentage of patients receiving ACT treatment. We also analyzed the relation between the total number of included *Plasmodium* infection cases per hospital and the percentage of intensive care or intermediate care hospitalizations and did not find any statistically significant relation (data not shown).

### *potra* Sequencing and Analysis

In total, 49 *potra* genes were sequenced from *P. ovale wallikeri* and 41 *potra* genes were sequenced from *P. ovale curtisi*. Three different genotypes (299, 317, and 335 bp) were identified in *P. ovale curtisi* and 4 different genotypes (245, 263, 263′, and 281 bp) in *P. ovale wallikeri* (Table 4). The major genotypes were (MANPIN)_1_(AITPIN)_2_ for *P. ovale wallikeri* and (TINPIN)_3_(TITPIS)_1_ for *P. ovale curtisi*. No association was found between country of contamination and *potra* genotype.

**Table 4:** Analysis of the *potra* fragment polymorphisms sequenced for *Plasmodium ovale wallikeri* and *P. ovale curtisi*, France, January 2013–December 2018

Species	Size, bp	Dominant amino acid repeat	No. (%) samples	GenBank accession no. of reference sequence
*P. ovale wallikeri*	245	(MANPIN)_1_(AITPIN)_2_	43 (88)	HMG594180
	263	(MANPIN)_1_(AITPIN)_3_	2 (4)	MG588149
	263	(MANPIN)_2_(AITPIN)_2_	1 (2)	MG588148
	281	(MANPIN)_2_(AITPIN)^3^	3 (6)	MG588150
*P. ovale curtisi*	299	(TINPIN)_3_(TITPIS)_1_	26 (63)	MG588152
	317	(TINPIN)_3_(TITPIS)_2_	13 (32)	HM594183
	335	(TINPIN)_4_(TITPIS)_2_	2 (5)	MG588154

## Discussion

Our findings show that patients infected with *P. ovale wallikeri* displayed deeper thrombocytopenia than those infected with *P. ovale curtisi* (p<0.001) and had a shorter latency period (p<0.001). Those features of *P. ovale wallikeri* infection are currently debated in the literature, with some studies describing deeper thrombocytopenia (*11*,*12*) and shorter latency periods (*9*) and other finding refuting any differences between the 2 species (*31*).

We reported 1.2% of patients with diagnosed *P. ovale* infection having severe criteria of malaria (*26*), a similar percentage to the data reported by the malaria surveillance in the United States (*32*) or by Kotepui et al. (*33*). Seven *P. ovale wallikeri*– and 1 *P. ovale curtisi*–infected patients were hospitalized in intensive or intermediate care. Six of those patients did not have WHO-defined severe malaria criteria (*26*). Hospitalization in intensive or intermediate care for non–WHO-defined severe malaria was previously described in uncomplicated malaria patients with *P. falciparum* (*34*) or *P. vivax* (*35*) infections. We examined the hospitalization information of 5,227 uncomplicated malaria patients (all infected with *Plasmodium* species) for the study period in the NMRC database. Among these patients, 180 (3.6%) were hospitalized in intensive or intermediate care with a median length of hospital stay shorter to that observed with severe malaria patients (median 2 days [IQR 1–3 days] vs. median 3 days [IQR 2–4 days]; p<0.001).

In June 2017, La Société de Pathologie Infectieuse de Langue Française updated malaria management recommendations (*4*) and proposed the use of ACT as first-line treatment for all *Plasmodium* spp. infections and placed atovaquone/proguanil as a second-line treatment. Our data confirmed that physicians followed the new guidelines with a clear change between ACT and atovaquone/proguanil prescription frequency (Figure 5). *P. ovale wallikeri* infections were treated more often with ACT. To explain this phenomenon, we compared the antimalarial treatment used according to the platelet counts, parasite density, pLDH-RDTs results, and latency period duration. No association was observed between the type of antimalarial treatment and platelet counts, parasite density, or pLDH-RDTs results, but we highlighted a relationship between ACT treatment and shorter latency period (p = 0.048). The combination of low platelet count and short latency delay in *Plasmodium* infections are suggestive of *P. falciparum* infection (*36*). In the context of emergency care before species confirmation, those features might have influenced the prescription of ACT. Because they were seen more frequently in *P. ovale wallikeri* infections, we assumed that this tendency could partially explain that most of the ACT treatment administered occurred in the *P. ovale wallikeri* group.

About 44% of patients that took a prophylactic treatment reported taking their medication regularly, as prescribed. The latency period was longer in those patients (p<0.001). Because prophylactic treatments are not effective against liver-dormant forms of *P. ovale* (*2*) and did not protect patients from relapsing malaria, those results are not surprising. This phenomenon is well-illustrated in military patients, a population with a higher rate of chemoprophylaxis treatment (85%) and greater compliance with the drug regimen (62%) who had longer latency periods than other patients (p<0.001).

Most of the *P. ovale* cases we analyzed were originally diagnosed by microscopic analysis. Species misidentification occurred for 8.8% of the samples, and the main misidentification was between *P. malariae* and *P. ovale*. In endemic settings, microscopic analysis or PCR diagnosis are not always available in remote setting. Simple and affordable point-of-care compatible diagnostic tools are required. Although RDTs are widely spread nowadays in malaria-endemic countries, their efficiency for *P. ovale* diagnosis is not sufficiently studied compared with that for *P. falciparum* of *P. vivax* diagnosis. To supplement this deficiency, we analyzed the ability of aldolase and pLDH-RDTs to detect *P. ovale wallikeri* and *P. ovale curtisi* infection (Table 2). Aldolase-RDTs detection was definitively more accurate for *P. ovale* diagnosis than pLDH-RDTs (p<0.001). pLDH-RDTs used in this study (Palutop+4 [Biosynex] and Core Malaria [Core Diagnostics, https://www.corediagnostics.net]) were more efficient in diagnosing *P. ovale wallikeri* than *P. ovale curtisi* infection, but their performance remained extremely low (≈16% of infections diagnosed). This discrepancy might be explained by lactate dehydrogenase protein polymorphisms in *P. ovale* (*37*) affecting affinity of RDT-antibodies for *P. ovale* lactate dehydrogenase (*38*). Tang et al. (*39*) compared the efficiency of several pLDH-RDTs and confirmed variable diagnostic performance for *P. ovale*. In contrast, aldolase-RDTs had similar efficiency in detection of both species (50% for *P. ovale wallikeri* and 41.2% for *P. ovale curtisi*) that increased with parasite density (Table 2; Figure 4). Vikia demonstrated better performances than BinaxNow in *P. ovale* spp. detection (p<0.001).

The ability of *P. ovale* to establish liver-dormant forms (hypnozoïtes) induces relapse episodes of fever and parasitemia (*2*,*40*). Relapsing malaria was observed in only 3.5% of the included patients, a lower prevalence than previously reported (*14*). This difference is probably linked to the recommendations in France that advises systematic primaquine treatment of all *P. ovale*–infected patients, even for the first episode (except for major contraindication such as G6PD deficiency, pregnancy, and breastfeeding) (*4*). Currently, diagnosis of *P. ovale* infection relapse is mainly based on clinical data. *potra* gene sequencing has been used to distinguish reinfection from relapse by genotyping the initial and corresponding relapse sample (*13*,*14*). We evaluated the polymorphism of *potra* genes in 80 samples and, as previously described, identified a limited number of polymorphisms (Table 4) (*28*). Our results, combined with those of Zhou et al. (*29*), demonstrate that the *potra* gene is not a satisfying genetic marker of relapse. New genetic markers, such as microsatellite typing, need to be developed for *P. ovale* genotyping, as was previously done for *P. falciparum* (*41*,*42*) and *P. vivax* (*43*,*44*).

A limitation of our study is that, because of uncompleted online patient form filling (Appendix Table 2), we might lack statistical power to highlight differences in some rare infections features, such as hospitalization in intensive or intermediate care. In addition, our study is retrospective and might suffer from missing data about infection characteristics. Furthermore, we collected *P. ovale *isolates from Africa only.

In conclusion, our large retrospective study on *P. ovale wallikeri* and *P. ovale curtisi* infections confirmed that patients infected with *P. ovale wallikeri* display deeper thrombocytopenia and shorter latency periods. In addition, we found that physicians in France used more ACT to treat *P. ovale wallikeri* than *P. ovale curtisi* infections. This difference might be linked to the lower platelet level and shorter latency period seen with *P. ovale wallikeri* infections. In addition, we described a higher in intensive or intermediate care admission in *P. ovale wallikeri*–infected patients. Because of missing data and lack of power, this observation was not statistically significant and needs to be confirmed by a large, prospective study.

Appendix Additional information about *Plasmodium ovale wallikeri* and *P. ovale curtisi* infections and diagnostic approaches to imported malaria, France, 2013–2018.
