# Supplementary material for: Plasmodium ovale wallikeri and P. ovale curtisi Infections and Diagnostic Approaches to Imported Malaria, France, 2013–2018
Source: Emerg Infect Dis. 2021 Feb;27(2):372–84. doi: 10.3201/eid2702.202143 (PMC7853592; doi:10.3201/eid2702.202143)
Supplement: Appendix — Additional information about Plasmodium ovale wallikeri and P. ovale curtisi infections and diagnostic approaches to imported malaria, France, 2013–2018. [file 20-2143-Techapp-s1.pdf]

Article DOI: https://doi.org/10.3201/eid2702.202143

Plasmodium ovale wallikeri and *P. ovale curtisi* Infections and Diagnostic Approaches to Imported Malaria, France, 2013–2018

Appendix

Appendix Table 1. No. *Plasmodium ovale curtisi* and *Plasmodium ovale wallikeri* infections, by country of contamination

Country of contamination	*Plasmodium ovale curtisi*, n = 309	*Plasmodium ovale wallikeri*, n = 368
Angola	1	0
Benin	5	5
Burkina Faso	6	3
Cameroon	53	74
Central African Republic	22	25
Chad	4	1
Congo	11	18
Democratic Republic of Congo	4	5
Equatorial Guinea	3	0
Gabon	7	9
Ghana	1	3
Guinea	30	20
Ivory Coast	89	118
Kenya	0	1
Mali	11	13
Mauritania	1	0
Mozambique	0	1
Niger	1	1
Nigeria	6	10
Rwanda	0	1
Senegal	3	7
Sierra-Leone	2	3
Tanzania	0	2
Togo	12	14
Two or more visited countries	27	23
Missing data	10	11

Appendix Table 2. Number of missing data

Characteristic	No. (%) *P. ovale curtisi*, n = 309	No. (%) *P. ovale wallikeri*, n = 368
Age	0 (0)	0 (0)
Sex	0 (0)	0 (0)
Ethnicity	40 (12.9)	52 (14.1)
If African, place of birth	27 (15.6)	34 (16.6)
Duration of travel	118 (38.2)	132 (35.9)
Type of patient	90 (29.1)	108 (29.3)
Chemoprophylaxis	66 (21.4)	55 (14.9)
Bed nets using	131 (42.4)	165 (44.8)
Parasite density	2 (0.6)	2 (0.5)
Leucocytes	25 (8.1)	32 (8.7)
Hemoglobin	22 (7.1)	28 (7.6)
Platelets	20 (6.5)	33 (9)
Diagnostic delay	58 (18.8)	82 (22.3)
Delay between return from endemic country and onset of symptoms	86 (27.8)	102 (27.7)
Symptoms		
Fever	35 (11.3)	45 (12.2)
Arthralgia/Myalgia	90 (29.1)	129 (35.1)
Asthenia	128 (41.4)	151 (41)
Headache	89 (28.8)	101 (27.4)
Type of malaria	9 (29.1)	24 (6.5)
Admission to hospital	25 (8.1)	43 (11.7)
Duration of hospitalization	71 (44.9)	101 (51.5)
Type of hospitalization	111 (70.3)	134 (68.3)
Treatment	38 (12.3)	48 (13)
Country of contamination	10 (3.2)	11 (3)

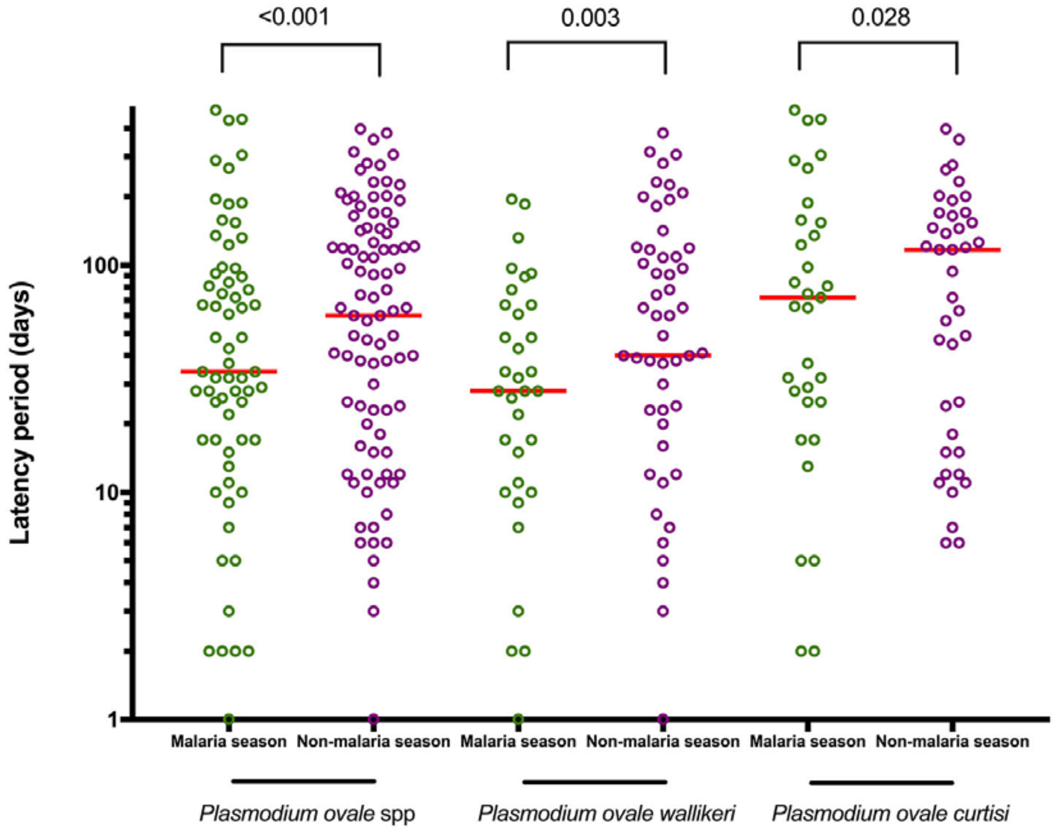

Appendix Figure. Latency period (days) of infections contracted in West Africa for *Plasmodium ovale* spp., *Plasmodium ovale wallikeri*, and *Plasmodium ovale curtisi* according to the season.
